# Lanbotulinumtoxin‐A: Safe for children with cerebral palsy but is it effective?

**DOI:** 10.1111/dmcn.15329

**Published:** 2022-06-23

**Authors:** Tandy Hastings‐Ison, Kerr Graham

**Affiliations:** ^1^ Department of Physiotherapy, Melbourne School of Health Sciences University of Melbourne Melbourne Australia; ^2^ Orthopaedic Department Royal Children's Hospital Parkville Victoria Australia

## Abstract

This commentary is on the original article by Li et al. on pages 86‐93 of this issue.

Adverse events are a key issue when considering the prescription of medications for children with cerebral palsy. The study by Li et al. is the first large study of adverse events following injection of Lanbotulinumtoxin‐A in children with cerebral palsy (CP). The rate of adverse events following injection of Lanbotulinumtoxin‐A was similar to that following injection of Onabotulinumtoxin‐A (aka Botox) at 12% versus 14% respectively.^1^ Most adverse events were mild and self‐limiting. Risk factors for adverse events were higher Gross Motor Function Classification System level, history of recent illness, and total dose of injected neurotoxin.^1^ The animal studies referenced by Li et al. compared the effects of injecting the rat medial gastrocnemius muscle, with four different type A toxins to injection with normal saline.^2^ The injection of any one of the four type A toxins, including both Lanbotulinumtoxin‐A and Onabotulinumtoxin‐A, resulted in comparable muscle atrophy, which had not resolved at 12 weeks post‐injection. ^2^ The take‐home message is that type A toxins are similar in both clinical effects and side effects.

These findings are important because Lanbotulinumtoxin‐A is significantly cheaper than Onabotulinumtoxin‐A. The costs of botulinum neurotoxin‐A have posed a significant restraint on the use of the neurotoxin in many countries. The availability of a less‐expensive neurotoxin‐A product with a comparable safety profile is potentially good news for children with CP and their parents/guardians who may pay for toxin injections in some jurisdictions, as well as for global healthcare funding systems looking to constrain or reduce pharmaceutical costs. However, this simple conclusion is not based on clinical outcomes data.^3^

Clinically relevant outcome measures were not included in this study by Li et al., who investigated adverse events in isolation. Current literature shows that it is becoming as difficult to find a black cat in a dark room as to demonstrate objective, clinically verifiable, positive outcomes from botulinum toxin therapy in children with CP.^3^, ^4^

The most frequently injected muscles sites in the Li et al. study were gastrocnemius and soleus.^1^ In a recent study from our centre investigating objective kinematic changes in children with spastic equinus following botulinum toxin injections to these muscle groups, we were unable to demonstrate clinical improvements in Gait Profile Score.^4^

In a more recent study, Schwartz et al. estimated the short‐term effectiveness of 13 common orthopaedic and neurological treatments at four different levels of outcome in children with CP.^5^ The outcome measures addressed body structures, specific gait kinematic deviations, overall gait kinematic deviations, and functional mobility. There were two interventions which had large ‘average treatment effects on the treated’ at the body structures level. These were selective dorsal rhizotomy and femoral osteotomy. For most common treatments, there was diminishing impact as one proceeded down the causal chain, from body structure to specific gait to global gait to functional mobility. The models used in this elegant study found no effect of neurotoxin injections in any of the four domains.

When outcomes are subjective, such as goal attainment scaling, or if spasticity is inappropriately linked as a causal factor in activity or participation restrictions, the benefits of neurotoxin injections may be overestimated. When objective gait laboratory measures are used, the benefits of neurotoxin injection are undetectable or insignificant.^4^, ^5^ Perhaps the black cat has left the room, or more disturbingly, was never there in the first place?

This study confirms a comparable safety profile between Lanbotulinumtoxin‐A and Onabotulinumtoxin‐A for children with CP. As Lanbotulinumtoxin‐A provides a cheaper alternative, it may become more widely available. This is an opportunity to design robust studies with objective, verifiable, and clinically meaningful outcomes. ^4^, ^5^

## Data Availability

Not required.
